# The Role of Social Support vs. Modeling on Adolescents' Diet and Physical Activity: Findings from a School-based Weight Management Trial

**DOI:** 10.4172/2375-4494.1000132

**Published:** 2014-04-30

**Authors:** Monica Wang, Susan Druker, Mary Ann Gapinski, Lauren Gellar, Kristin Schneider, Stavroula Osganian, Barbara Olendzki, Lori Pbert

**Affiliations:** 1 Division of Preventive and Behavioral Medicine, University of Massachusetts Medical School, 55 Lake Ave North, Worcester, MA 01655, USA; 2 Department of Social and Behavioral Sciences, Harvard School of Public Health, 677 Huntington Ave., Boston, MA 02115, USA; 3 Massachusetts Department of Public Health, 250 Washington St., Boston, MA 02108, USA; 4 Nutrition Department, University of Tennessee, 1215 W. Cumberland Ave., Knoxville TN 37996, USA; 5 Department of Psychology, Rosalind Franklin University of Medicine and Science, 3333 Green Bay Rd., North Chicago, IL 60064, USA; 6 Department of Medicine, Children’s Hospital Boston, 333 Longwood Ave., Boston, MA 02115, USA

**Keywords:** Obesity, Adolescent, Social support, Modeling, Diet, Physical activity

## Abstract

**Objective:**

Social influences play an important role in shaping adolescents’ dietary and physical activity behaviors. We examined the role of perceived modeling and perceived social support from family and friends on diet and physical activity behaviors among overweight and obese adolescents participating in a weight management trial.

**Methods:**

Six high schools were randomized to a school-nurse delivered behavioral weight management intervention or an information attention-control. Data on perceived support and modeling of healthy eating and physical activity from family and friends and dietary and physical activity behaviors were obtained from participants (N=82) at baseline and 2- and 6-months follow-up.

**Results:**

Linear mixed models were used to examine associations between social factors at baseline and diet and physical behaviors at 6 months. Friend support was correlated with increased fruit and vegetable consumption (0.4 servings/day) and decreased added sugar intake (−14.2 grams/day) (p’s<0.05). Family support for physical activity, friend support for physical activity, and family modeling of physical activity were associated with increased number of days/week active for ≥ 60 minutes/day (0.7 days/week; 0.6 days/week; and 0.4 days/week, respectively, p’s<0.05).

**Conclusions:**

Among overweight and obese high school adolescents, support from family and friends was associated with a greater number of improvements in diet and physical activity at follow-up than modeling. Strategies to solicit support may maximize efficacy of adolescent obesity intervention efforts.

## Introduction

Adolescence is a critical period for addressing overweight and obesity, with 15.2% of U.S. adolescents in grades 9-12 overweight and 13.0% obese in 2011 [1]. Obesity during adolescence is associated with immediate and long-term negative health outcomes, including heightened risk of developing type 2 diabetes, hypertension, depression, and continued obesity into adulthood [2,3]. Healthy weight management among adolescents is critical, particularly as they establish independence and make more choices outside of the home environment [4], with respect to diet and physical activity behaviors.

Schools serve as a valuable intervention setting for obesity intervention, yet less than 14% of school-based interventions targeting body mass index (BMI) took place in high schools (approximate student population age range of 14-18 years) [5,6]. Findings from a pilot cluster-randomized trial of a school-based weight management intervention targeting high school youth indicated that the intervention was associated with improvements in dietary behaviors, but not BMI or physical activity, among overweight and obese adolescents [7]. Understanding the interplay of other factors related to weight management, such as social norms and influences, is essential for advancing obesity intervention efforts among this population and preventing obesity-related complications and conditions later on in the life course.

Social support for behavior change and modeling of targeted behaviors are important behavior change facilitators [8]. Identifying how modeling and support influence diet and physical activity is critical, as these experiences often occur outside of the intervention context. Several studies indicate the importance of parental and peer modeling of eating and physical activity patterns and social support for weight management efforts as important factors associated with children’s dietary and physical activity patterns, particularly among younger children [9-15]. However, the influence and provision of social support and modeling from various sources may change across child development. For example, as children transition into adolescence, peer norms and behaviors may become more important in shaping diet and physical activity. The examination of factors that may predict differential treatment outcomes among older adolescents is important, given the relative lack of school-based interventions targeting this age group. To address this gap, this study aimed to examine modeling and social support from family and friends associated with changes in diet and physical activity over time among overweight and obese high school adolescents participating in a pilot school-based weight management trial.

## Methods

### Participants and procedures

Data from this study were from a cluster-randomized trial of a school-nurse delivered weight management intervention targeting overweight and obese adolescents [7]. Six high schools in Massachusetts were pair-matched on size and racial/ethnic composition, with one school from each pair randomly assigned to a school-nurse delivered counseling intervention or an information control condition. Participant eligibility included: age- and sex-adjusted BMI at or above the 85th percentile, provision of assent and parental consent to participate in the study; and having at least one English-speaking parent. Exclusion criteria included having a medical condition that precluded adherence to the intervention; diagnosis of a serious psychiatric illness; genetic or endocrine cause of obesity; taking a medication associated with weight gain; or weighing ≥ 300 pounds. Participants were recruited through school announcements, flyers and posters, and health-related school nurse encounters.

The intervention, “Lookin’ Good Feelin’ Good”, consisted of six one-on-one patient-centered counseling sessions delivered by school nurses to adolescent participants in the school nurse office over 2 months. The Social Cognitive Theory-based intervention targeted adolescents only (no family involvement) and included cognitive behavioral techniques to facilitate changes in diet and physical activity. Height and weight measurements were obtained by school nurses at each visit. Participants in control schools had six one-on-one school nurse visits over a 2-month period; these visits included height and weight measurement, review of diet and physical activity behavioral changes, and overview on weight management information. Measures were collected at baseline, 2 months, and 6 months follow-up from 2008-2009. Further details regarding the intervention and study eligibility, recruitment, screening, and procedures have been previously described [7,16]. All study procedures were approved by the University of Massachusetts Medical School Human Subjects Institutional Review Board.

### Measures

Measures for this study were selected from those included in the original study. For perceived social support, participants reported the extent to which two sources (family members and friends) helped them “eat healthy foods” and/or “be physically active” using a 4-point Likert scale (Cronbach α=0.70; test-retest r=0.66-0.73) [17]. Higher scores indicated greater support for the behavior indicated. Perceived modeling of behaviors was assessed using modified social norms items related to fruit and vegetable consumption [18]; participants reported the number of family members and the number of friends who “eat healthy” and/or who “are physically active.”

Dietary intake was assessed with a single 24-hour dietary recall interview [19] using the interactive Nutrition Data System for Research (NDSR, version 2008, annually updated) and conducted on a randomly selected day. Dietary behaviors targeted by the intervention (fruit servings/day, vegetable servings/day, and number of times consuming the following on a typical day over the past 7 days: breakfast, fast food, and non-diet soda servings) were assessed using a validated 8-item instrument [20]. The number of days that adolescents were active for at least 60 minutes/day over the past 7 days (a recommended guideline and targeted intervention behavior) [21,22] was used to assess physical activity.

Gender, race (White, Black/African American, Asian, American Indian/Alaskan Native, Other), ethnicity (Hispanic/Latino), and free and reduced price lunch program participation (yes/no) were assessed using self-administered student questionnaires.

### Statistical analysis

Descriptive statistics were conducted to examine predictors, covariates, and outcomes of interest at baseline. Linear mixed effects regression models were conducted to assess the unique contribution of baseline measures of social support and modeling associated with dietary and physical activity behavior change (baseline to 6 months), adjusting for intervention status and time. Schools were included as a random effect in models to account for within-school clustering of observations. Analyses were conducted using SAS version 9.3.

## Results

Of the 82 study participants (mean age of 15.8 years), the majority were female (70%) and White (77%). At baseline, intervention (n=42) and control participants (n=40) differed with respect to participation in the free and reduced price lunch program and soda consumption. See Table 1 for additional details on baseline characteristics. All participants completed follow-up measures.

Table 2 presents findings of the associations between various social influences at baseline and dietary and physical activity outcomes at 6 months follow-up among all study participants, controlling for intervention status and time. Friend support to eat healthy was positively associated with fruit consumption (servings/day) (β=0.2, 95% Cl: 0.03 to 0.5) and fruit and vegetable consumption (servings/day) (β=0.4, 95% CI: 0.02 to 0.8) and negatively associated with added sugar intake (grams/day) (β=−14.2, 95% CI: −25.6 to −2.8). These associations remained significant when family support to eat healthy was added to the model, although family support to eat healthy was not independently associated with dietary outcomes. Thus, only friend support to eat healthy was included in the final model.

Family support to be physically active, friend support to be physically active, and number of family members who were physically active were each positively associated with the number of days adolescents were active for at least 60 minutes/day (0.7, 95% CI: 0.4 to 1.03; 0.6, 95% CI: 0.3 to 0.9; 0.4, 95% CI: 0.07 to 0.7, respectively) and percent of adolescents who were active for at least 5 days in the past 7 days (0.2, 95% CI: 0.1 to 0.3; 0.1, 95% CI: 0.04 to 0.2; 0.9, 95% CI: 0.01 to 0.2, respectively). When both family and friend support to be physically active were included in the model, neither were significant.

## Discussion

Findings from this school-based trial indicated that perceived social support from family and friends at baseline was associated with several positive changes in diet and physical activity behaviors at follow-up among overweight and obese adolescents, although different patterns emerged depending on behavior. Friend support to eat healthy was associated with higher fruit and vegetable intake and lower added sugar intake, whereas family support to eat healthy and family and friend modeling of healthy eating were not associated with dietary behaviors. These findings contrast with previous reviews indicating that family support and encouragement to eat healthy, in particular from parents, and family behaviors, such as parental modeling of healthy eating habits generally predict healthy dietary behaviors among children and adolescents [23,24], though the limited nature of the measures of social influences (only four items measuring adolescent perception of support and modeling for healthy eating and physical activity) may partially account for these findings. A possible explanation for this finding is that as older adolescents establish independence (typically spending less meal-time with parents and more meal-time with peers than younger children, and having greater control over food purchasing and intake than younger children), family members may exert less influence over dietary behaviors while peers may be more influential among older adolescents. An alternative explanation is that adolescents may experience deficits in parental support for healthy eating compared to younger children. Additional longitudinal research on change in family support for healthy eating and other behaviors (perceived by children and reported by parents) over time is needed and may inform future school-based and family-based obesity interventions.

Within the physical activity behavioral domain, baseline measures of perceived family and friend support for physical activity and perceived family modeling of physical activity were associated with increased physical activity at 6 months; these results are consistent with existing studies documenting parental and peer influences on children’s physical activity [9,10,25]. However, no changes in BMI associated with the intervention were reported in the original study [7]. Findings from the current study suggest that exploring a variety of strategies to increase family and friend support for physical activity, family modeling of physical activity, and friend support to eat healthy may useful in facilitating healthy weight management behaviors for overweight and obese adolescents.

Results from this study offer preliminary indications that helping adolescents solicit support from key members of their social network may facilitate behavior change for weight management. For adolescents who may lack support from in-person relationships, social media and internet-based programs can serve as potential mechanisms to obtain support [26]. A study comparing sources of support from in-person (family members and friends) and online relationships (Facebook friends and Twitter followers) among adults trying to lose weight indicated that online friends provided as much positive social support for physical activity as family and in-person friends [27]. The adoption and implementation of a recent school-based internet obesity intervention targeting a multiethnic sample of high school students indicated high student satisfaction and participation [5]. These findings highlight the potential of harnessing social media and incorporating internet-based components for obesity interventions among adolescents, a group that has high interent and social media usage. Future research will be needed to determine the best mediums (technology-based and non-technology-based) for social support and modeling of healthy diet and physical activity among adolescents.

Study strengths include the examination of the role of support and modeling from two referent groups among high school youth, an under-studied age group with respect to school-based interventions. Limitations included the correlational nature of the study, restricted measures of predictors and outcomes, self-report measurement of most measures (thus, responses may be biased by social desirability), and lack of generalizability of findings due to the small sample size recruited from one geographic region.

### Implications for research and practice

Social support for healthy eating and physical activity is correlated with positive diet and physical activity behaviors among overweight and obese adolescents. School-based or family-based obesity interventions should consider incorporating messages and/or strategies, including internet-based and social media components, for adolescents to solicit support to maximize the beneficial effects of the intervention. Studies that systematically compare the influence of social support and modeling of healthy eating and physical activity and assess change in the provision of social support (e.g., type, amount) from various referent groups on children’s diet and physical activity at different developmental stages, particular mid-to-late adolescence, are needed.

## Figures and Tables

**Table 1:** Baseline Characteristics of Overweight and Obese Adolescents (N=82) Participating in a School-Based Cluster Randomized Controlled Trial Targeting Weight Management.

	Intervention (N=42)	Control (N=40)	p-value*
Sociodemographics			
Age	15.9 (1.03)	15.7 (1.01)	0.516
Gender (% Female)	64.3	75.0	0.343
Race/Ethnicity			0.435
% White	73.8	80.0	
% Black	14.3	5.0	
% Hispanic Ethnicity	14.3	15.0	
Participation in free or reduced price lunch (%)	47.6	17.5	0.005
BMI			
Mean BMI percentile	96.4 (3.4)	95.3 (3.8)	0.188
% 85th < BMI <95th percentile (age and sex-adjusted)	21.4	40.0	
% BMI > 95th percentile (age and sex-adjusted)	78.6	60.0	
Perceived family & friend support			
Family support to engage in healthy eating	2.9 (0.6)	2.7 (0.8)	0.277
Friend support to engage in healthy eating	2.0 (0.6)	2.1 (0.9)	0.213
Family support to be physically active	2.8 (0.6)	2.6 (0.8)	0.222
Friend support to be physically active	2.8 (0.8)	2.5 (0.9)	0.796
Perceived family & friend modeling			
# of family members who eat healthy	2.2 (0.8)	2.3 (0.7)	0.966
# of friends who eat healthy	2.0 (0.7)	2.2 (0.8)	0.366
# of family members who are physically active	2.1 (0.8)	2.1 (0.9)	0.845
# of friends who are physically active	2.6 (0.6)	2.5 (0.7)	0.700
Dietary behaviours			
Servings of fruit in a typical day in past 7 days			0.439
% None	4.8	7.5	
% 1-2 servings per day	42.9	55.0	
% 3 or more servings per day	52.4	37.5	
Servings of vegetables in a typical day in past 7 days			1.000
% None	9.5	7.5	
% 1-2 servings per day	50.0	50.0	
% 3 or more servings per day	40.5	42.5	
Servings of fruit and vegetables in a typical day in past 7 days			0.439
% None	4.8	7.5	
% 1-2 servings per day	42.9	55.0	
% 3 or more servings per day	52.4	37.5	
# of times drink soda (not diet) in a typical day in past 7 days			0.020
% None	19.1	37.5	
% 1-2 times per day	66.7	35.0	
% 3 or more times per day	14.3	27.50	
Mean # days eat breakfast in past 7 days	3.1(2.4)	3.7(2.9)	0.327
# of times eat food from fast food restaurant in past 7 days			0.257
% None	31.0	47.5	
% 1-2 times	54.8	37.5	
% 3 or more times	14.3	15.0	
Mean total sugar intake (grams)	109.6 (72.7)	116.4 (56.2)	0.644
Mean added sugar intake (grams)	73.7 (63.2)	79.0 (55.8)	0.501
Physical activity behaviours			
# of days active for at least 60 min. per day over past 7 days	4.8 (1.9)	4.0 (2.3)	0.0821
% active for ≥ 5 days over past 7 days	30.9	30.0	0.931

**Table 2:** Social Support and Modeling of Healthy Eating and Physical Activity Associated with 6-Month Changes in Diet and Physical Activity among Overweight and Obese Adolescents (N=82) Participating in a School-Based Weight Management Trial* *Models adjusted for intervention status, time, and clustering of individuals within schools; significant (p<0.05) effect estimates are bolded.

	Support to Eat Healthy		Behaviours (Eat Healthy)		Support to Be Physically Active		Behaviours (Are Physically Active)	
Dietary outcomes	Family Support	Friend Support	# of Family Members	# of Friends	Family Support	Friend Support	# of Family Members	# of Friends
Servings of fruit in a typical day in past 7 days	0.1 (−0.1, 0.4)	0.2 (0.03, 0.5)	0.1 (−0.1, 0.3)	0.008 (−0.2, 0.2)	--	--	--	--
								
Servings of vegetables in a typical day in past 7 days	0.2 (−0.04, 0.5)	0.2 (−0.07, 0.4)	0.1 (−0.1, 0.4)	0.2 (−0.03, 0.4)	--	--	--	--
								
Servings of fruit and vegetables in a typical day in past 7 days	0.4 (−0.06, 0.8)	0.4 (0.02, 0.8)	0.2 (−0.2, 0.6)	0.2 (−0.2, 0.6)	--	--	--	--
								
# of times drink soda (not diet) in a typical day in past 7 days	−0.2 (−0.4, 0.1)	0.02 (−0.2, 0.3)	−0.2 (−0.4, 0.07)	0.07 (−0.2, 0.3)	--	--	--	--
								
Mean # days eat breakfast in past 7 days	0.3 (−0.1, 0.7)	0.2 (−0.2, 0.6)	0.07 (−0.3, 0.5)	0.06 (−0.3, 0.4)	--	--	--	--
# of times eat food from fast food restaurant in past 7 days	−0.1 (−0.3, 0.08)	−0.1 (−0.3, 0.04)	−0.1 (−0.3, 0.05)	0.01 (−0.2, 0.2)	--	--	--	--
								
Mean total sugar intake (grams)	−6.2 (−19.1, 6.6)	−11.9 (−23.8, 0.07)	−6.9 (−19.8, 6.1)	−10.9 (−23.9, 2.1)	--	--	--	--
Mean added sugar intake (grams)	−10.3 (−22.5, 1.8)	−14.2 (−25.6, −2.8)	−11.4 (−23.6, 0.9)	−4.8 (−17.6, 8.0)	--	--	--	--
Physical activity outcomes								
# of days active for at least 60 min. per day in past 7 days	--	--	--	--	0.7 (0.4, 1.03)	0.6 (0.3, 0.9)	0.4 (0.07, 0.7)	0.08 (−0.3, 0.5)
% active for >5 days in past 7 days	--	--	--	--	0.2 (0.1, 0.3)	0.1 (0.04, 0.2)	0.9 (0.01, 0.2)	0.03 (−0.06, 0.1)
